# Quantification of Biogenic Amines in 35 Korean Cottage Industry Traditional *Gochujang* (Fermented Red Pepper Paste) Products

**DOI:** 10.3390/foods10102370

**Published:** 2021-10-06

**Authors:** Srinivasan Ramalingam, Ashutosh Bahuguna, SeMi Lim, Ah-ryeong Joe, Jong-Suk Lee, So-Young Kim, Myunghee Kim

**Affiliations:** 1 Department of Food Science and Technology, Yeungnam University, Gyeongsan 38541, Korea; sribt27@gmail.com (S.R.); ashubahuguna@gmail.com (A.B.); thfvkalfpeh7@naver.com (S.L.); whdkfud12@naver.com (A.-r.J.); 2 Division of Food & Nutrition and Cook, Taegu Science University, Daegu 41453, Korea; jslee1213@ynu.ac.kr; 3 Department of Agrofood Resources, National Institute of Agricultural Sciences, Rural Development Administration, Wanju 55365, Korea; foodksy@korea.kr

**Keywords:** fermentation, food safety, HPLC, histamine, principal component analysis, putrescine, red pepper paste

## Abstract

Traditional *gochujang* is well known for its distinguished flavor and taste. However, the safety of cottage industry *gochujang* products is uncertain, particularly, in terms of biogenic amine (BA) content which is not yet documented. The present study aimed to determine the level of BAs present in 35 traditional *gochujang* products nationwide. All *gochujang* products had considerable amounts of total BAs ranging from 52.95 mg/kg to 176.24 mg/kg. Individually, histamine and tyramine were either not detected or detected up to 16.94 mg/kg and 2.15–52.34 mg/kg, respectively. In all the tested *gochujang* products, putrescine, spermidine, and spermine were detected in the range of 7.60–56.72 mg/kg, 14.96–36.93 mg/kg, and 4.68–16.31 mg/kg, respectively. A total of 22 and 19 *gochujang* products had less than 1 mg/kg of cadaverine and histamine, respectively. The findings indicate that all the *gochujang* products tested herein had BA levels below the suggested toxicity limits recommended by the various regulatory authorities, which reveal that they are safe for human consumption.

## 1. Introduction

*Gochujang* (fermented red pepper paste) is a traditional Korean fermented food that is generally used as a sauce in Korean cuisines and as a seasoning in spicy foods. In 2017, the total domestic retail market revenue of *gochujang* was 183.63 billion Korean won (~USD 149.55 million). *Gochujang* products were also exported to many countries, including the US, Japan, and China, generating a total of USD 31.98 million in revenue [1]. *Gochujang* is prepared as a paste by mixing various ingredients, such as powdered red pepper (*Capsicum annuum* L.), saccharified grain starch (rice, wheat, or barley), salt, powdered *meju* (natural soybean-based starter), and potable water. The mixture is then fermented and aged [2]. The primary ingredients of *gochujang* include red hot pepper powder, *meju*, and rice powder. In general, *gochujang* is prepared using two different methods: (1) traditional homemade method and (2) large-scale production in quality-controlled industries [3,4].

Household and cottage *gochujang* industries rely on traditional fermentation techniques using simple equipment, where fermentation is influenced by the natural microflora present in *meju* and surrounding environmental factors. However, large-scale industries employ controlled environment conditions and pure starter cultures, such as *Aspergillus* and *Bacillus* species, to produce *gochujang* [5]. The *gochujang* cottage industry in different provinces generates products with diversified nutritional values and organoleptic properties. Raw ingredients, process methods, microorganisms involved in the fermentation, and duration of the fermentation markedly influence the organoleptic properties of *gochujang,* including its aroma, taste, and texture [5]. Nowadays, consumers are highly interested in indigenous branded traditional *gochujang* products owing to the consistent outstanding sensory quality. However, the safety of *gochujang* products from the cottage industry is uncertain. These traditional cottage *gochujang* industries have minimal capital, and thus cannot afford to establish a quality control analysis department as part of their industry. The physicochemical, microbial, and toxicological profiles of these *gochujang* products are not available to the public to ensure consumer safety as they are not frequently monitored either by the production companies or by any food and health organization. Furthermore, the quality of *gochujang* products generated in the indigenous branded cottage industry has not been sufficiently examined [6].

BAs are organic nitrogenous compounds with low molecular weight that are mainly found in fermented foods. These are also microbial decarboxylation products of amino acids and are considered to be toxicants and the anti-nutritional elements of food [7]. An excessively high number of BAs, such as histamine (>500 ppm), putrescine (>2000 ppm), spermine (>600 ppm), and spermidine (>600 ppm), induces considerable toxicity [7]. Notably, these nitrogenous compounds are mostly produced by microorganisms during fermentation through enzymatic decarboxylation of amino acids, as well as transamination of ketones and aldehydes [8]. Several studies have reported the presence of BAs in a wide range of food products, including fermented foods, different types of seafood, agricultural commodities [9], and baby foods, alcoholic beverages, and halal foods [10]. The number of BAs in different fermented foods varies and is associated with the type of available amino acids, the presence of BA-forming bacteria, and different factors that affect microbial activity, such as appropriate pH, temperature, water activity, and time [10]. During the fermentation of *gochujang*, the native microbes convert the protein-rich raw materials (soybean in the *meju*) into different types of BAs, such as histamine, tyramine, β-phenylethylamine, tryptamine, putrescine, and cadaverine, as a result of the putrefactive process of proteins [11].

BAs are essential for cellular development and growth and are important regulators of several processes, such as brain activity, body temperature regulation, stomach pH, gastric acid secretion, the immune response, and the synthesis of hormones and alkaloids [10]. However, the consumption of an excessive number of BAs leads to serious physiological and toxicological effects. Del Rio et al. [12] reported the harmful effect of BAs in boosting histamine toxicity, in addition to being responsible for the so-called “cheese reaction”. BAs cause different levels of intoxication in different individuals owing to variations in genetic predisposition and changes in the level of mono and di-amine oxidase enzymes in the intestinal epithelium. Mono and di-amine oxidase enzymes are sensitive to BAs and impair the functioning of the small intestine or kidneys. Accordingly, celiac patients, people undergoing surgery, or those receiving treatment for cancer and other pathologies are affected by BAs [10]. The most frequent BA-related food intoxication is caused by histamine poisoning, previously known as scombroid fish poisoning as it was first reported to be caused by the *Scombridae* family, such as tuna. Secondary amines, such as putrescine and cadaverine, also play an important role in food poisoning, as they can increase the toxicity of histamine or react with nitrites to form carcinogenic nitrosamines [13]. Excessive consumption of BAs leads to serious implications, such as nausea, headache, heart and respiratory diseases, hypotension, and hypertension [14]. Therefore, the assessment of BAs is essential to ensure the safety of fermented foods.

Recently, studies on the anti-cholesterol, anti-obesity, and anti-atherosclerotic properties of *gochujang* have gained immense attention worldwide. Previously, several studies focused on improving the quality of *gochujang* by assessing the effects of additives and microflora on the organoleptic properties [15,16,17]. Additionally, numerous investigations have focused on the storage methods for extending the shelf life of *gochujang*, including physical sterilization methods and the addition of natural food ingredients [18]. Similarly, few studies have reported the BAs content of the large-scale industries and lab-made/home-made *gochujang* samples [19,20,21]. However, to the best of our knowledge, the quantity of BAs in *gochujang* products from the cottage industry has not been determined in any of the previous studies. Given the importance of BAs in the safety of fermented foods, it is essential to determine its content and evaluate other safety protocols of *gochujang* products to ensure consumer safety and inform consumer choice of appropriate *gochujang* products. Thus, the present study aimed to determine the quantity of various BAs, such as agmatine, cadaverine, histamine, 2-phenylethylamine, putrescine, spermidine, spermine, tryptamine, and tyramine, in 35 *gochujang* products collected from nationwide cottage industries.

## 2. Materials and Methods

### 2.1. Chemicals

Sodium hydroxide, sodium hydrogen carbonate, ammonium hydroxide, and perchloric acid were purchased from Junsei Chemicals (Seoul, Korea). Dansyl chloride and standard BAs were purchased from Sigma-Aldrich (St. Louis, MO, USA). High-performance liquid chromatography (HPLC)-grade ammonium acetate and acetonitrile were purchased from Merck (Damstadt, Germany). All chemicals used in this study were of analytical grade and were used as supplied.

### 2.2. Sample Collection

A total of 35 *gochujang* products were purchased from cottage industries in different provinces of the Republic of Korea. The major ingredients of *gochujang* products include red pepper powder, glutinous rice powder, powdered *meju*, grain syrup, malt, salt, and water. The codes and ingredients for the selected *gochujang* product are presented in Table 1.

### 2.3. Estimation of BAs

The BAs present in the *gochujang* were estimated using the protocol of Ramalingam et al. [22]. In brief, 5 g samples were extracted twice with 25 mL of 0.4 M perchloric acid. Thereafter, the extracts were centrifuged at 4000× *g* for 10 min at 4 °C, filtered using a Whatman paper No. 1, and subjected to dansyl chloride derivatization. The extracts were mixed with 2 M NaOH (200 µL), a saturated solution of NaHCO_3_ (300 µL), and 0.04 M dansyl chloride (2 mL), and then incubated at 40 °C for 45 min. After the incubation period, 100 µL of 25% NH_4_OH was added to the reaction mixture to stop the reaction. The volume of the mixture was increased to 5 mL using acetonitrile, to remove excess dansyl chloride. This mixture was then centrifuged (2500× *g* for 5 min at 4 °C) and eventually filtered through a 0.2 µm membrane filter (Sartorius AG, Goettingen, Germany).

The BAs were quantified using an HPLC system (Thermo Fisher Scientific, Waltham, MA, USA) coupled with a UV-visible detector. A C18 column (5-µm pore size, 4.6 × 250 mm, XBridge Shield RP18, Waters Corporation, Milford, MA, USA) was used to separate the BAs by HPLC. In the HPLC program, the gradient flow rate of the mobile phase (0.1 M ammonium acetate and acetonitrile) was 1 mL/min for 35 min and the column isothermal temperature was 30 °C. The sample (20 µL) was injected by auto sample injection and analyzed at 254 nm. Standard curves were plotted for known standard BAs, which were used for sample quantification.

### 2.4. Statistical Analysis

All experiments were carried out at least in triplicate, and the values are presented as mean ± standard deviation. Statistical analyses were performed using SPSS software 23 (IBM, Chicago, IL, USA). One-way analysis of variance (ANOVA) in a completely randomized design and Duncan’s multiple range comparison tests were used to determine significant differences between the samples with a 95% confidence limit at *p* < 0.05. The multivariate exploratory techniques of principal component analysis (PCA) were carried out to categorize the *gochujang* samples based on their quantities of BAs using Xlstat package on Microsoft Office Excel 2016 version.

## 3. Results and Discussion

*Gochujang* fabricated in the traditional cottage industry contains a mixture of complex raw materials (including *meju,* a protein rich substrate), which are fermented by different microorganisms and are thus more vulnerable to BA production [1]. Histamine, tyramine, cadaverine, 2-phenylethylamine, spermine, spermidine, putrescine, tryptamine, and agmatine are the predominant BAs present in fermented foods [7]. Typically, excess BAs in foods are produced by the action of microorganisms. Therefore, the level of BAs in foods is an indirect measure of the microbial load [7]. As BAs are one of the main toxicants in fermented foods, we assessed their presence in 35 *gochujang* products from different cottage industries found nationwide and their impact on the quality and safety of the products.

**Figure 1:**
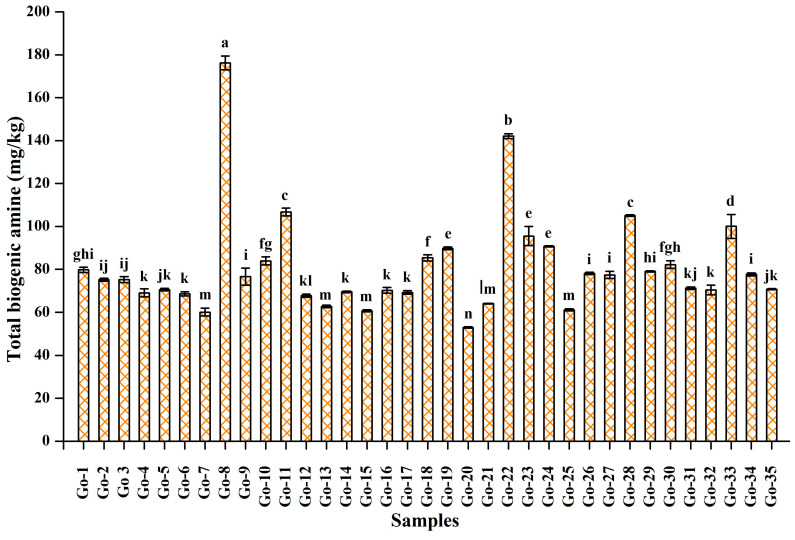
Total biogenic amines quantity in 35 *gochujang* products from the cottage industry. Different lowercase letters on the bars indicate significant differences (*p* < 0.05) based on Duncan’s multiple comparison test.

The total BAs in all *gochujang* samples ranged from 52.95 mg/kg to 176.24 mg/kg. The highest amount of BA was observed in Go-8 (176.24 ± 3.24 mg/kg), followed by Go-22 (142.11 ± 1.12 mg/kg), Go-28 (105.05 ± 0.36 mg/kg), Go-11 (106.76 ± 1.85 mg/kg), and Go-33 (100.03 ± 5.55 mg/kg), whereas the least amount was detected in Go-20 (52.95 ± 0.32 mg/kg) (Figure 1). Based on the total BAs, the *gochujang* samples were further classified into three categories: group I, not detected level to 75 mg/kg BAs; group II, 75–100 mg/kg BAs; and group III, greater than 100 mg/kg BAs. The majority of *gochujang* samples (45.71%) fell in group I, followed by group II (40%) and group III (14.28%). Although there are no strict guidelines for total BAs in fermented food, many reports suggest 1000 mg/kg as the maximum permissible limit [23]. The variation in BA content in the different *gochujang* products is due to several reasons; however, the most evident is the different raw materials used to prepare the products. Microbial population and physiochemical conditions also significantly contributed to the variations. The *gochujang* samples tested herein had BAs within the suggested limit (<1000 mg/kg), and the results were consistent with those of published reports, demonstrating a similar type of BA profile in various *gochujang* samples [19,20,21]. The low amount of the BAs in the *gochujang* samples might be due to excellent hygienic conditions, selection of raw materials, and fermentation environments, which are the major factors that influence the production of BAs.

**Figure 2:**
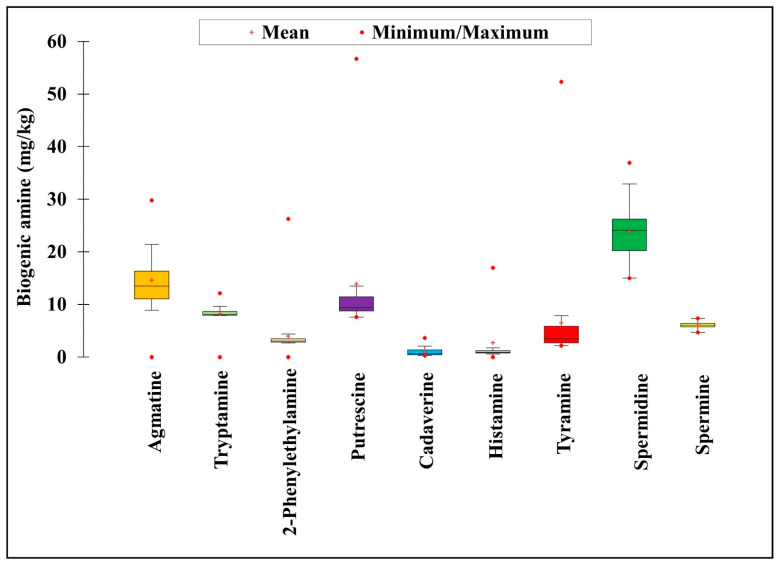
Mean quantity of various biogenic amines in the 35 traditional cottage industry *gochujang* products.

Among the different BAs, histamine has garnered more attention due to its severe toxic effect (scombroid poisoning) [7]. Therefore, most regulatory bodies, such as the US Food and Drug Administration (US FDA) and the European Food Safety Authority, emphasize the histamine level in foods. According to the US FDA guidelines, 50 mg/kg histamine is the legal limit for fishes [24], whereas the European Commission has set 100 mg/kg as the permissible limit in fishes and 400 mg/kg in fish sauces produced by fermentation [25]. The World Health Organization also suggested 200 mg/kg as the maximum permissible limit of histamine in food products [26]. Similarly, the regulatory authorities of Korea and China suggested 200–400 mg/kg of histamine as the maximum permissible limit in food products [27,28]. In the 35 *gochujang* products tested herein, histamine levels ranged from not detectable to 16.94 mg/kg (Figure 2 and Table 2). The maximum amount of histamine was detected in Go-28 (16.94 ± 0.03 mg/kg), whereas the least amount was detected in Go-25, Go-27, and Go-32. Based on the histamine level, the samples were divided into three categories: group I, no detection level to 5 mg/kg histamine; group II, 5–10 mg/kg histamine; and group III, >10 mg/kg histamine. Most of the *gochujang* samples (82.86%) were assigned to group I, followed by group III (11.43%) and group II (5.71%). The *gochujang* samples Go-1 (10.36 ± 0.17 mg/kg), Go-11 (11.27 ± 0.02 mg/kg), Go-28 (16.94 ± 0.03 mg/kg), and Go-33 (15.54 ± 2.16 mg/kg) had histamine levels >10 mg/kg. Nonetheless, the histamine level in all samples was below the suggested toxicity limit recommended by various regulatory authorities, suggesting that the 35 *gochujang* products were safe for consumption based on their histamine levels. The results align well with those of Lee et al. [21] and Cho et al. [19], thereby highlighting a similar type of histamine level in different *gochujang* samples. However, Kim et al. [20] reported slightly higher histamine levels (2.2–59.0 mg/kg) in eight *gochujang* samples.

Tyramine is a concerning BA in food, is responsible for the cheese reaction, and causes syndromes, such as histamine poisoning [29]. The regulatory authorities have not provided strict guidelines for tyramine. However, few reports suggest that the toxic limit of tyramine is 100–800 mg/kg [23,30]. Herein, tyramine was detected to range from 2.15–52.34 mg/kg in the tested *gochujang* samples (Figure 2 and Table 2); these levels are below the recommended toxicity limit suggested by the regulatory authorities [23,30]. The highest amount of tyramine was detected in Go-8 (52.34 ± 0.41 mg/kg), whereas the least amount was in Go-7 (2.15 ± 0.03 mg/kg). Based on the level of tyramine, we further divided the *gochujang* samples into three groups: group I, no detection level to 5 mg/kg tyramine; group II, 5–20 mg/kg tyramine; and group III, >20 mg/kg tyramine. Most *gochujang* samples were assigned to group I (65.71%), followed by group II (28.57%) and group III (5.71%). The level of tyramine in the samples was below the toxicity level of 100–800 mg/kg [23,30] and aligned with the levels found by Kim et al. [20] in 8 *gochujang* samples (range of 2.9–126.8 mg/kg).

Agmatine is another concerning BA in foods that is formed by the decarboxylation of L-arginine and is readily converted into putrescine [31,32]. In the *gochujang* samples, agmatine was either not detected or detected up to 29.78 mg/kg, with the highest amount found in Go-22 (29.78 ± 0.12 mg/kg) (Figure 2 and Table 2). Based on the detected amount of agmatine, the samples were divided into three groups: group I, not detected level to 10 mg/kg agmatine; group II, 10–20 mg/kg agmatine; and group III, >20 mg/kg agmatine. Most of the *gochujang* samples were assigned to group II (68.57%), followed by group III (17.14%) and group I (14.28%).

Putrescine, spermidine, and spermine are common BAs found in foods. The active role of spermidine and spermine as food allergens has been reported by several researchers [33,34,35]. To date, no legal limit has been recommended for putrescine, spermidine, and spermine; however, many studies suggest oral toxicity limits of 2000 mg/kg, 600 mg/kg, and 600 mg/kg, respectively [7,36]. In the 35 *gochujang* samples assessed herein, putrescine, spermidine, and spermine were detected in the range of 7.60–56.72 mg/kg, 14.96–36.93 mg/kg, and 4.68–16.31 mg/kg, respectively (Figure 2 and Table 2). The maximum amount of putrescin was detected in Go-8 (56.72 ± 0.50 mg/kg), whereas the least amount was detected in Go-21 (7.60 ± 0.01 mg/kg). The maximum amount of spermidine (36.93 ± 0.20 mg/kg) and spermine (16.31 ± 0.04 mg/kg) was detected in Go-19 and Go-5, respectively, whereas the least amount of spermidine (14.96 ± 0.02 mg/kg) and spermine (4.68 ± 0.01 mg/kg) was detected in Go-20. These findings are consistent with the reports that demonstrated variations in these BAs in different *gochujang* samples [19,20]. Lee et al. [21] determined the presence of BAs in eight *gochujang* products and found that the amount of putrescine and spermidine ranged 0.3–2.6 mg/kg and 0.6–1.3 mg/kg, respectively. Unlike our results, Lee et al. [21] detected a lower amount of putrescine and spermidine in the *gochujang* samples tested. This variation might be due to different raw materials used and the involvement of microbial population during the fermentation of *gochujang*.

2-Phenylethylamine is one of the most frequently found BAs in foods and together with other BAs, provokes food-induced migraine and hypertension [37]. There is no legal limit for 2-phenylethylamine in food samples; however, few independent researchers suggested 30 mg/kg as the toxic threshold [30]. Herein, 2-phenylethylamine was either not detected or detected up to 26.23 mg/kg in the *gochujang* samples, with the highest amount found in Go-22 (26.23 ± 0.08 mg/kg; Figure 2 and Table 2). These results align well with those of previous studies [19,20,21], thereby indicating that the content of 2-phenylethylamine varies in different *gochujang* samples. Kim et al. [20] tested *gochujang* samples and found 1.8–24.8 mg/kg of 2-phenylethylamine, which is consistent with the present findings. Compared with the present study, Lee et al. [21] found a lower amount of 2-phenylethylamine (range of 0.5–2.0 mg/kg).

Cadaverine and tryptamine are other frequently found BAs in foods; however, no legal limit has been established for cadaverine and tryptamine in foods. Cadaverine has no significant toxicity; however, its presence synergistically enhances the toxic effect of histamine. Cadaverine also has a negative impact on the catabolism of histamine by interfering with histamine detoxification [38,39,40]. Herein, the level of cadaverine ranged from 0.31–3.63 mg/kg in the *gochujang* samples, with the highest level found in Go-11 (3.63 ± 0.02 mg/kg) (Figure 2 and Table 2). The results align well with those of Cho et al. [19] and Kim et al. [20], showing the similar presence of cadaverine in different *gochujang* samples. The involvement of tryptamine in increasing blood pressure, which can lead to hypertension has been well reported in the literature [41]. Therefore, its presence in food is critical to the establishment of food safety. Tryptamine was either not detected or detected up to 12.11 mg/kg in different *gochujang* samples, with the highest amount detected in Go-24 (12.11 ± 0.05 mg/kg) (Figure 2 and Table 2). Among the 35 *gochujang* products, 8.57% of the products had >10 mg/kg of tryptamine. Such findings aligned well with those of Lee et al. [21], who determined tryptamine levels in eight *gochujang* products. However, the amount of tryptamine (0.1–4.1 mg/kg) in the *gochujang* products tested in their study was lower than that detected in the products tested in the present study.

All analyzed *gochujang* products harbored low quantity of BAs. Major factors that affect the quantity of BAs in traditional *gochujang* products include manufacturing practice, ratio and quality of raw material, the microbial consortium involved during fermentation, fermentation environment, and the duration of the fermentation process. The predominant bacterial species found in *gochujang* are *Bacillus subtilis,* followed by *Bacillus licheniformis* and *Bacillus amyloliquefaciens,* which are well known producers of BAs due to the presence of decarboxylase enzyme [42,43]. The decarboxylase activities in these bacteria markedly vary between the strains and may be a major cause of the difference in the quantity of BAs in various *gochujang* products [10]. Besides bacteria, some studies reported that yeast can also produce BAs. Particularly, *Zygosaccharomyces rouxii* is predominant in traditional *gochujang*. However, yeasts have been considered to produce only negligible amounts of BAs [13,44].

A considerably high number of BAs was found in Go-22, followed by Go-28, which contains soybean, *cheonggukjang* (a fast fermented soybean paste), and *meju* which provide a protein rich environment for the production of BAs. Go-8, Go-22, and Go-28 also had higher amounts of free amino nitrogen (data not shown), which may influence the formation of BAs in those products as they are the readily available substrates for the synthesis of various types of BAs. In general, red pepper is the major ingredient in traditional *gochujang* products [1]. Red pepper contains capsaicin as a bioactive compound, which has been evaluated for its inhibitory effect against the formation of BAs [7]; this might be one of the reasons for the low production of BA in *gochujang* products. Moreover, *gochujang* has an acidic pH and high salinity, which are known to affect the production of BA and control the growth of BA producing microbes [7]. Further, numerous strains of *Bacillus* sp. isolated from the different fermented foods are found to actively degrade BAs [13], which supports the low amount of BA in *gochujang* products owing to the predominance of these species in traditional *gochujang* products. The additives (for example, alcohol added in G-20 *gochujang* product) mixed with *gochujang* may also play a role in the reduction of BAs either by decreasing BA production or inhibiting the growth of BAs producing microbes [7].

The overall results indicated that the mean and median values of the estimated BAs, namely agmatine, tryptamine, 2-phenylethylamine, putrescine, cadaverine, histamine, tyramine, spermidine, and spermine in *gochujang* products from the traditional cottage industry were 14.60, 8.24, 3.96, 13.88, 0.98, 2.72, 6.47, 23.95, and 6.09 mg/kg, and 13.48, 8.04, 3.03, 9.37, 0.57, 0.82, 3.45, 24.09, and 6.00 mg/kg, respectively (Figure 2). Similarly, the minimum and maximum amounts of putrescine, cadaverine, tyramine, spermidine, and spermine in the *gochujang* samples were 7.60, 0.27, 2.15, 14.96, and 4.68 mg/kg, and 56.72, 3.63, 52.34, 36.93, and 7.31 mg/kg, respectively. Agmatine, tryptamine, histamine, and 2-phenylethylamine were either not detected or detected up to maximum amounts of 29.78, 12.11, 16.94, and 26.23 mg/kg, respectively, in the *gochujang* samples. Although few samples had 100–175 mg/kg of total BA, the average total BA content in the *gochujang* samples was 80.09 mg/kg, which is quite lower than the toxic limit set by many government entities and independent researchers. The present results indicate that all tested *gochujang* products from the traditional cottage industry are safe for consumption in terms of BA content.

**Figure 3:**
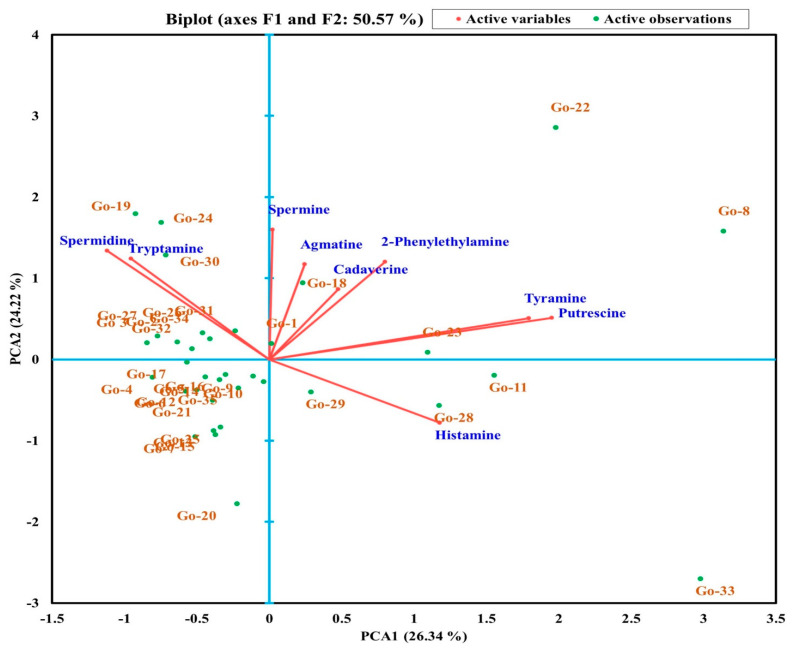
Principal component analysis of biogenic amines in different *gochujang* products from the traditional cottage industry.

Multivariate analysis using PCA was performed to segregate the BAs in different *gochujang* samples (Figure 3). PC1 separated Go-1, Go-8, Go-11, Go-18, Go-22, Go-23, Go-28, and Go-29, (positive PC1 values) from the other samples (Figure 3). Further, PC2 explained the variance and separated Go-4, Go-6, Go-7, Go-9, Go-12, Go-13, Go-15, Go-16, Go-20, Go-21, Go-23, Go-25, Go-28, Go-29, and Go-35 from the other samples (Figure 3). PCA separated and derived the different clusters of 35 *gochujang* samples based on their BAs. The histamine content significantly influenced Go-29, Go-28, and Go-11 *gochujang* products, whereas the spermine, agmatine, cadaverine, 2-phenylethylamine, tyramine, and putrescine content influenced Go-1, Go-18, Go-23, Go-22, and Go-8. However, a few of the *gochujang* samples did not show any variation in BA quantities (Figure 3). The spermidine and tryptamine contents displayed significant variance with the other tested BAs, and maximum variance was found between histamine and spermidine, followed by tryptamine. To the best of our knowledge, no study has categorized the BAs in *gochujang* samples using multivariate PCA; however, a few studies have used PCA to represent the BA profiling in food products [45,46].

## 4. Conclusions

All the *gochujang* products from the cottage industry had markedly lower levels of BAs than the suggested toxicity limits, indicating good manufacturing practice, excellent hygienic conditions, and the selection of high-quality raw material for *gochujang* preparation. The major reason for low levels of BAs in the analyzed traditional *gochujang* might be the use of raw materials containing low proteinaceous substances, except *meju* (fermented soybeans). This study revealed the safety of tested traditional *gochujang* produced by the cottage industry in different provinces of Korea. The results also revealed that the levels of BAs in the cottage-based traditional *gochujang* products posed no issue of toxicity, thereby indicating that these products are safe for human consumption.

## Figures and Tables

**Table 1:** Details of the selected *gochujang* products.

Product Code	Location of the Company (in the Republic of Korea)	Ingredients
Go-1	Jindo-gun, Jeollanam-do	garlic, meju * powder
Go-2	Chungju, Chungcheongbuk-do	soybean, apple extract
Go-3	Cheongyang-gun, Chungcheongnam-do	red pepper powder, sea salt, maesil **
Go-4	Jeongeup-si, Jeollabuk-do	red pepper powder, sea salt, *meju* powder
Go-5	Yesan-gun, Chungcheongnam-do	jujube cherry tomato puree, apple enzyme, grain syrup, red pepper powder, sea salt, yeast
Go-6	Yesan-gun, Chungcheongnam-do	rice grain syrup, apple enzyme, red pepper powder, *meju*, salt, yeast
Go-7	Eumseong-gun, Chungcheongbuk-do	red pepper powder, shiitake mushroom, soybean, salt
Go-8	Daegu-si	red pepper powder, *maesil* sugar syrup, garlic, salt
Go-9	Sunchang-gun, Jeollabuk-do	red pepper powder, sea salt, glutinous rice, malt
Go-10	Sunchang-gun, Jeollabuk-do	red pepper powder, sea salt, barley, malt
Go-11	Sunchang-gun, Jeollabuk-do	red pepper powder, sea salt, *maesil*, malt
Go-12	Sunchang-eup, Jeollabuk-do	glutinous rice, *meju* powder, rice flour, malt, sea salt
Go-13	Sunchang-eup, Jeollabuk-do	glutinous rice, sea salt, malt, *maesil* sugar syrup
Go-14	Sunchang-eup, Jeollabuk-do	red pepper powder, glutinous rice, *meju* powder, rice flour, malt, sea salt
Go-15	Sunchang-gun, Jeollabuk-do	red pepper powder, glutinous rice, sea salt, malt, *meju* powder, *maesil* sugar syrup
Go-16	Sunchang-gun, Jeollabuk-do	red pepper powder, glutinous rice, sea salt, malt, *meju* powder
Go-17	Sunchang-gun, Jeollabuk-do	red pepper powder, barley, sea salt, malt, *meju* powder
Go-18	Iksan-si, Jeollabuk-do	germinated brown rice, red pepper powder, malt, rice grain syrup
Go-19	Gangjin-gun, Jeollanam-do	red pepper powder, loofah enzyme, sea salt
Go-20	Hamyang-gun, Gyeongsangnam-do	red pepper powder, malt, rice grain syrup, glutinous rice, bamboo salt, alcohol
Go-21	Hamyang-gun, Gyeongsangnam-do	glutinous rice, malt, red pepper powder, bamboo salt
Go-22	Seocheon-gun, Chungcheongnam-do	soybean, sea salt, glutinous rice flour, red chili powder
Go-23	Seoul	traditional *meju*, glutinous rice flour, malt
Go-24	Sunchang-gun, Jeollabuk-do	red pepper powder, glutinous rice, *meju* powder, rice flour, malt, sea salt
Go-25	Hamyang-gun, Gyeongsangnam-do	red pepper powder, glutinous rice, malt, *meju* powder, sea salt
Go-26	Yecheon-gun, Gyeongsangbuk-do	red pepper powder, *meju* powder, sea salt
Go-27	Iksan-si, Jeollabuk-do	*sancho* *** pepper powder, *meju* powder, sea salt, barley powder, *maesil* sugar syrup, glutinous rice powder
Go-28	Bucheon-si, Gyeonggi-do	red pepper powder, *meju* powder, cheonggukjang **** powder, barley, malt
Go-29	Haenam-gun, Jeollanam-do	red pepper powder, glutinous rice, rice grain syrup, cider, shochu *****, *meju* powder, sugar
Go-30	Pocheon-si, Gyeonggi-do	red pepper powder, glutinous rice, rice grain syrup, malt, *meju* powder, sea salt
Go-31	Bonghwa-gun, Gyeongsangbuk-do	red pepper powder, glutinous rice, *meju* powder, malt, sea salt
Go-32	Pyeongchang-gun, Gangwon-do	red pepper powder, *meju* powder, glutinous rice powder, *maesil* sugar syrup, malt, rice grain syrup
Go-33	Gunsan-si, Jeollabuk-do	red pepper powder, rice, sea salt, *meju* powder, malt
Go-34	Jangheung-gun, Jeollanam-do	red pepper powder, sea salt
Go-35	Gongju-si, Chungchungnam-do	red pepper powder, glutinous rice, sea salt, soybean, malt

*Meju* *: Brick of fermented soybean produced by pounding, kneading, and shaping the cooked soybeans followed by a long period of fermentation in the natural or conditioned environment; *Maesil* **: *Prunus mume* Siebold and Zucc; *Sancho* ***: *Zanthoxylum piperitum* (L.) DC.; *Cheonggukjang* ****: Fast fermented soybean paste, produced by fermenting the boiled soybeans with *Bacillus subtilis* for 2–4 days; *Shochu* *****: A Korean alcoholic beverage containing 16.9–19% ethanol.

**Table 2:** Quantity of different biogenic amines (mg/kg) in the 35 *gochujang* products from the traditional cottage industry.

Samples	Agmatine	Tryptamine	2-Phenylethylamine	Putrescine	Cadaverine	Histamine	Tyramine	Spermidine	Spermine
Go-1	8.92 ± 0.34	7.95 ± 0.00	2.67 ± 0.00	10.13 ± 0.18	1.42 ± 0.11	10.36 ± 0.17	3.69 ± 0.11	27.47 ± 0.17	7.19 ± 0.03
Go-2	8.89 ± 0.14	8.51 ± 0.02	2.90 ± 0.02	9.78 ± 0.03	0.75 ± 0.04	0.77 ± 0.04	6.91 ± 0.09	30.37 ± 0.36	6.32 ± 0.01
Go 3	10.60 ± 0.10	10.35 ± 0.16	2.70 ± 0.01	10.25 ± 0.29	0.68 ± 0.01	0.60 ± 0.20	5.56 ± 0.15	28.62 ± 0.58	5.88 ± 0.07
Go-4	10.73 ± 0.92	9.90 ± 0.06	3.04 ± 0.04	9.07 ± 0.04	0.47 ± 0.01	1.19 ± 0.01	2.92 ± 0.17	26.03 ± 0.49	5.67 ± 0.17
Go-5	9.99 ± 0.19	8.10 ± 0.01	2.73 ± 0.01	10.06 ± 0.06	0.35 ± 0.01	1.71 ± 0.02	5.36 ± 0.10	25.90 ± 0.16	16.31 ± 0.04
Go-6	12.25 ± 0.36	7.97 ± 0.01	2.71 ± 0.02	8.30 ± 0.02	0.39 ± 0.00	0.77 ± 0.10	4.67 ± 0.04	26.69 ± 0.36	5.76 ± 0.46
Go-7	9.24 ± 0.06	7.90 ± 0.01	3.36 ± 0.09	8.61 ± 0.07	0.27 ± 0.01	0.54 ± 0.00	2.15 ± 0.03	22.48 ± 1.40	5.47 ± 0.12
Go-8	19.60 ± 0.90	8.90 ± 0.02	4.35 ± 0.08	56.72 ± 0.50	0.99 ± 0.03	0.81 ± 0.00	52.34 ± 0.41	24.16 ± 1.29	6.39 ± 0.01
Go-9	14.45 ± 1.85	7.92 ± 0.00	4.60 ± 1.60	9.51 ± 0.29	0.56 ± 0.03	4.32 ± 0.06	5.00 ± 0.02	24.09 ± 0.06	6.12 ± 0.01
Go-10	15.86 ± 0.92	7.97 ± 0.00	3.08 ± 0.00	10.94 ± 0.35	0.57 ± 0.01	5.47 ± 0.30	7.82 ± 0.02	26.36 ± 0.10	5.81 ± 0.00
Go-11	10.66 ± 1.12	7.88 ± 0.00	4.46 ± 0.03	24.24 ± 0.10	3.20 ± 0.16	11.27 ± 0.02	17.05 ± 0.30	22.36 ± 0.09	5.59 ± 0.01
Go-12	13.13 ± 0.41	7.92 ± 0.01	2.72 ± 0.00	9.07 ± 0.03	0.38 ± 0.12	1.18 ± 0.02	2.75 ± 0.02	24.57 ± 0.24	6.00 ± 0.01
Go-13	11.87 ± 0.40	7.91 ± 0.10	2.98 ± 0.01	8.50 ± 0.02	0.39 ± 0.00	1.19 ± 0.07	2.94 ± 0.07	21.35 ± 0.03	5.53 ± 0.01
Go-14	15.58 ± 0.22	7.89 ± 0.00	2.83 ± 0.00	8.69 ± 0.00	1.37 ± 0.01	0.86 ± 0.00	3.02 ± 0.02	23.48 ± 0.05	5.75 ± 0.04
Go-15	11.35 ± 0.37	7.90 ± 0.00	2.74 ± 0.00	8.92 ± 0.02	0.41 ± 0.00	0.76 ± 0.01	2.70 ± 0.01	2.70 ± 0.03	5.58 ± 0.02
Go-16	15.61 ± 1.24	7.90 ± 0.00	2.93 ± 0.00	9.22 ± 0.02	1.44 ± 0.01	0.77 ± 0.03	3.19 ± 0.00	23.28 ± 0.02	5.82 ± 0.01
Go-17	15.08 ± 0.25	7.92 ± 0.00	2.81 ± 0.00	7.63 ± 0.00	0.41 ± 0.01	1.06 ± 0.01	2.59 ± 0.06	25.33 ± 0.17	6.37 ± 0.32
Go-18	25.54 ± 0.10	8.04 ± 0.01	3.16 ± 0.02	11.23 ± 0.07	2.03 ± 0.00	0.72 ± 0.02	7.81 ± 0.15	19.87 ± 0.67	6.94 ± 0.08
Go-19	16.75 ± 0.09	10.37 ± 0.06	3.08 ± 0.05	8.68 ± 0.00	3.63 ± 0.02	1.09 ± 0.07	2.89 ± 0.04	36.93 ± 0.20	6.30 ± 0.09
Go-20	11.44 ± 0.23	7.89 ± 0.01	2.92 ± 0.01	7.72 ± 0.01	0.31 ± 0.01	0.72 ± 0.03	2.28 ± 0.00	14.96 ± 0.02	4.68 ± 0.01
Go-21	15.52 ± 0.03	8.04 ± 0.02	2.84 ± 0.00	7.60 ± 0.01	0.31 ± 0.01	1.16 ± 0.01	2.28 ± 0.00	20.18 ± 0.09	6.05 ± 0.01
Go-22	29.78 ± 0.12	8.04 ± 0.00	26.23 ± 0.08	31.42 ± 0.04	1.58 ± 0.02	0.82 ± 0.01	14.90 ± 0.16	22.46 ± 0.55	6.85 ± 0.14
Go-23	ND *	8.06 ± 0.07	8.47 ± 0.71	41.61 ± 4.42	1.16 ± 0.06	0.72 ± 0.06	6.05 ± 0.48	20.28 ± 0.36	6.61 ± 0.08
Go-24	21.16 ± 0.05	12.11 ± 0.05	2.85 ± 0.02	13.30 ± 0.06	0.84 ± 0.03	1.20 ± 0.02	5.23 ± 0.01	26.80 ± 0.01	7.24 ± 0.02
Go-25	12.25 ± 0.34	7.89 ± 0.00	3.43 ± 0.02	9.25 ± 0.03	0.55 ± 0.08	ND *	2.43 ± 0.00	19.76 ± 0.02	5.55 ± 0.01
Go-26	21.02 ± 0.13	7.96 ± 0.00	3.03 ± 0.00	9.37 ± 0.01	0.38 ± 0.01	0.74 ± 0.00	3.45 ± 0.02	25.76 ± 0.32	6.41 ± 0.01
Go-27	15.23 ± 0.11	8.84 ± 0.28	3.50 ± 0.02	8.65 ± 0.17	1.56 ± 0.21	ND *	3.11 ± 0.13	31.03 ± 1.35	5.49 ± 0.06
Go-28	16.78 ± 0.20	9.24 ± 0.01	4.84 ± 0.02	24.39 ± 0.03	0.56 ± 0.00	16.94 ± 0.03	6.72 ± 0.03	19.65 ± 0.03	5.89 ± 0.01
Go-29	13.48 ± 0.01	8.46 ± 0.00	3.33 ± 0.00	13.47 ± 0.03	0.66 ± 0.01	8.75 ± 0.14	4.38 ± 0.02	20.02 ± 0.01	6.44 ± 0.03
Go-30	13.08 ± 0.03	8.78 ± 0.02	4.11 ± 1.32	11.56 ± 0.08	1.06 ± 0.01	0.69 ± 0.01	3.72 ± 0.06	32.89 ± 0.08	7.31 ± 0.12
Go-31	14.41 ± 0.30	8.08 ± 0.01	2.82 ± 0.01	8.82 ± 0.08	2.93 ± 0.01	0.69 ± 0.03	3.05 ± 0.02	24.25 ± 0.11	6.15 ± 0.02
Go-32	13.04 ± 0.84	8.08 ± 0.12	2.93 ± 0.06	10.55 ± 0.54	0.44 ± 0.02	ND *	2.61 ± 0.25	24.93 ± 1.19	6.66 ± 0.13
Go-33	10.67 ± 0.23	ND *	ND *	30.50 ± 3.01	1.31 ± 0.13	15.54 ± 2.16	20.23 ± 2.21	16.35 ± 0.07	5.39 ± 0.03
Go-34	25.71 ± 0.50	9.59 ± 0.04	3.31 ± 0.01	9.11 ± 0.07	0.53 ± 0.01	1.07 ± 0.04	2.24 ± 0.01	20.09 ± 0.00	5.99 ± 0.01
Go-35	21.43 ± 0.06	7.97 ± 0.01	3.98 ± 0.07	8.88 ± 0.03	0.49 ± 0.01	0.74 ± 0.02	2.22 ± 0.02	19.27 ± 0.06	5.73 ± 0.01

ND *: not detected.

## Data Availability

Data is contained within the article.
